# Experienced Homophobia and Suicide Among Young Gay, Bisexual, Transgender, and Queer Men in Singapore: Exploring the Mediating Role of Depression Severity, Self-Esteem, and Outness in the Pink Carpet Y Cohort Study

**DOI:** 10.1089/lgbt.2020.0323

**Published:** 2021-06-30

**Authors:** Rayner Kay Jin Tan, Timothy Qing Ying Low, Daniel Le, Avin Tan, Adrian Tyler, Calvin Tan, Chronos Kwok, Sumita Banerjee, Alex R. Cook, Mee Lian Wong

**Affiliations:** ^1^ Saw Swee Hock School of Public Health, National University of Singapore and National University Health System, Singapore, Singapore.; ^2^ Faculty of Arts and Social Sciences, National University of Singapore, Singapore, Singapore.; ^3^ Action for AIDS Singapore, Singapore, Singapore.

**Keywords:** gay men, homophobia, Singapore, suicide

## Abstract

***Purpose:*** No prior study has been published on suicide-related behaviors among gay, bisexual, transgender, and queer (GBTQ) men in Singapore, where sexual relations between men are criminalized. This study explores the association and mediational pathways between experienced homophobia and suicidal ideation or suicide attempts among young GBTQ men in Singapore.

***Methods:*** Results of this study were derived from baseline data of the *Pink Carpet Y Cohort Study*, Singapore's first prospective cohort study among young GBTQ men. The sample comprised 570 young GBTQ men 18 to 25 years of age who were HIV negative or unsure of their HIV status. Statistical analyses were conducted through descriptive statistics, multivariable logistic regression, and structural equation modeling techniques.

***Results:*** Of 570 participants, 58.9% (*n* = 308) reported ever contemplating suicide, whereas 14.2% (*n* = 76) had ever attempted suicide. Controlling for key demographic variables, multivariable logistic regression revealed that experienced homophobia and depression severity were positively associated with a history of suicidal ideation, whereas depression severity and outness were positively associated with a history of suicide attempts. Mediation analyses revealed that depression severity and self-esteem partially accounted for the relationship between experienced homophobia and suicidal ideation, whereas depression severity and outness partially accounted for the relationship with suicide attempts.

***Conclusions:*** The prevalence of suicidal ideation and past suicide attempts was found to be high in a sample of young GBTQ men in Singapore. Interventions to address experienced homophobia and discrimination among young GBTQ men are needed urgently in Singapore.

## Introduction

The World Health Organization reported that the global crude suicide mortality rate was 10.6 per 100,000 persons in 2016.^1^ Based on the report, men (13.5) had higher mortality rates attributable to suicide than women (7.7), whereas residents in European (15.4) and Southeast Asia regions (13.2), as well as higher-income countries (14.3) reported higher suicide mortality rates compared with the rest of the world and those in lower- to middle-income countries, respectively.^1^ However, these findings should be interpreted in light of other factors such as suicide surveillance and reporting capacities, as well as other sociocultural factors that cannot be generalized across regions.

Sexual minority youths are more likely to exhibit suicide-related behavior (e.g., ideation, planning, attempting, and medically serious attempts) than their heterosexual counterparts.^2^ Suicidal ideation refers to thoughts of suicide without necessarily amounting to making plans for suicide, whereas suicide planning typically involves making a plan to do so.^3^ On the other hand, suicide attempts are differentiated from deaths in that the former does not result in the death of the person who attempted suicide. In two meta-analyses, Miranda-Mendizábal et al. found that young gay and bisexual men were more likely to attempt suicide when compared with heterosexual men,^4^ while di Giacomo et al. noted that transgender youths had greater risk of attempted suicide than bisexual and “homosexual” youths.^5^ In the United States, a study utilizing data from the National Violent Death Reporting System found that among individuals 12 to 29 years of age who died by suicide, those who were lesbian female, bisexual female, or transgender male were more likely to have died with a history of suicide attempts, whereas those who identified as gay male, lesbian female, bisexual male, bisexual female, or transgender female were more likely to have died with a history of suicidal thoughts, compared with “non-LGBT males.”^6^

A study in China noted that male adolescents who reported same-sex romantic attraction or both-sex romantic attraction were more likely to report past-year suicidal ideation and suicide attempts than their counterparts who identified as heterosexual.^7^ Another study in South Korea found that male youths who reported experiences of same-sex or both-sex sexual intercourse were more likely to report a history of suicidal ideation, suicide planning, and suicide attempts compared with those with opposite-sex sexual intercourse experiences only,^8^ suggesting that greater risk of suicide for sexual minority youths is not specific to Western developed societies.

In general, factors that are associated with increased risk of suicide in studies of LGBT and questioning (LGBTQ) individuals, as well as men who have sex with men (MSM), include mental health factors, such as substance use and poorer mental wellbeing.^6,8–13^ These include depressive symptoms, anxiety, and panic disorders that have generally been found to be associated with suicide-related behaviors.^14^ Furthermore, a history of peer victimization, homophobic bullying, or having experienced sexual violence have been found to be associated with both suicidal ideation and suicide attempts among LGBTQ youth in a variety of settings.^9,10,15^ With regard to social contexts, a study in Taiwan found that low levels of family support during childhood were associated with recent suicidality, or any suicidal ideation or attempted suicide, among gay and bisexual men,^16^ whereas another study in the United States found that higher levels of school belonging were associated with lower levels of suicidal ideation among LGBTQ youth.^17^

Some studies have noted that there are different risk factors for suicidal ideation and suicide attempts. For example, Mu et al. noted that mental disorders, such as depression, only increase the odds for suicidal ideation while other mental disorders related to general anxiety and panic disorders only increase the odds for suicide attempt among MSM in China.^12^ The authors also noted that disclosure of sexual identity increases the risk for suicidal ideation, but having their homosexual behavior known by family members increases the risk of suicide attempts.^12^ However, there has been a lack of consensus on which risk factors would be specific for suicidal ideation or suicide attempts. For example, the same study also noted that drug dependence raises the risk of suicide attempts but not suicidal ideation;^12^ however, this finding may contradict other studies which note that drug use increases both the risk of suicidal ideation and suicide attempts.^9,10^

It is estimated that ∼3.7% (*n* = 210,000) of Singapore's population of 5.7 million are MSM, based on a recent size estimation study.^18^ Although Singapore has made tremendous economic progress since its independence, Singapore society still holds largely negative views of sexual minority men. Nationally representative studies conducted in the past decade found that most Singaporeans were not in favor of the repeal of Section 377A, the colonial-era law that criminalizes sexual relations between men with penalty of imprisonment for a term which may extend to 2 years.^19–21^ Section 377A does not criminalize sex between women, and the Government of Singapore has adopted a nonenforcement policy of the law in private, consensual situations, while still retaining it as a symbol of conservative values of Singaporean society. However, individuals have been charged under this law upon complaints or reports where such incidents involved nonconsent, public acts of indecency, or minors.^19^

Apart from structural stigma toward sexual minority men caused by the criminalization of same-sex sexual behavior, past studies have also found that Singaporeans in general hold negative views toward same-sex behavior. A nationally representative survey conducted in 2013 by the Institute of Policy Studies in Singapore found that ∼80% of Singaporeans thought that sexual relations between two adults of the same sex were either “Always Wrong” or “Almost Always Wrong.”^20^ However, a more recent survey published in 2019 by the same institute showed that this figure had dropped to 63.6%, which they attributed to less conservative views among younger cohorts of Singaporeans and among those with higher education attainment.^20,22^ In recent years, the issue of gay rights has also garnered more attention in the public sphere with gay-friendly movements, such as Pink Dot Singapore, a yearly event supporting the freedom to love, gaining greater momentum since its initial run in 2009. This is, however, also accompanied by a growing countermovement in the form of the Wear White Campaign espousing pro-family values.^23^ Social stigma in Singapore toward sexual minority individuals has a negative impact on sexual health-related behavior among gay, bisexual, queer, and other MSM in Singapore.^24–26^

The aims of this study are twofold. First, it attempts to fill the gap in our knowledge of how experienced homophobia impacts the mental health of young gay, bisexual, transgender, and queer (GBTQ) men in Singapore, and thus allow for the development of appropriate interventions. Second, our inquiry into the mediating roles of depression severity, self-esteem, and outness was guided by minority stress theory.^27^ Specifically, the minority stress model describes how minority stressors, such as sexual orientation-based victimization or experienced homophobia contribute to physical and mental health problems.^27^ As such, we hypothesize that minority stressors, such as experienced homophobia, may lead to suicide-related behaviors through mental health comorbidities or covariates. We thus attempt to explore how mediating factors, including depression severity, self-esteem, and outness to others, may account for the relationship between experienced homophobia and suicide-related behaviors in young GBTQ men, especially in a setting where considerable stigma and the criminalization of sexual relations between men prevail.

## Methods

### Participants and recruitment

Ethics approval was obtained from the Institutional Review Board at the National University of Singapore (NUS-IRB Reference Code S-19-007) before data collection. Data for this study were derived from the *Pink Carpet Y Cohort Study*, Singapore's first prospective cohort among young GBTQ men 18 to 25 years of age and a collaboration between a nongovernmental organization Action for AIDS Singapore (AFA), and the Saw Swee Hock School of Public Health at the National University of Singapore and National University Health System (SSHSPH). To be eligible for this cohort, participants had to report being HIV negative or unsure of their HIV status; between the ages of 18 to 25 years; Singapore citizens or permanent residents; and identify in terms of sexual orientation as gay, bisexual, or queer; as well as in terms of gender identity as cisgender male (assigned gender at birth is male, gender identity is male), transgender male (assigned female at birth, gender identity is male), or queer men (assigned male at birth, do not identify with any particular gender now) at the point of recruitment across May to September 2019.

Participants were recruited through promotional flyers by a network of community-based organizations in Singapore who are engaged in health advocacy-related activities for young GBTQ men. These flyers were distributed both online and at physical venues. Participants who were interested in participating and were eligible for the study signed up through an enrolment web link embedded in these flyers with their self-reported alias, contact details, date of birth, gender, HIV status, sexual orientation, and their residence status. A copy of this enrolment survey is included in Supplementary Table S1 alongside a flow diagram describing the derivation of the analytic sample (Supplementary Fig. S1). The researchers ensured that no staff member from AFA or SSHSPH had full access to either the enrolment details held by AFA, which contained aliases and contact details of participants, and the baseline survey results held by SSHSPH. Upon clicking on or visiting the enrolment link, participants were led to a page where the participant information sheet was embedded, which they could download and keep. Participants who agreed to participate in the survey provided informed consent by clicking on a button at the end of the page to acknowledge that they have read the participant information sheet and agreed to participate in the survey.

Both sets of data were only linked by the unique identifier, which participants entered at the beginning of the survey. Upon completion of the survey, an SSHSPH staff member provided AFA with the unique identifiers of those who had completed the baseline survey, and an SGD 20.00 (approximately USD 15.00) cash reimbursement was given to the participant. Participants could also refer their friends to participate in the survey and be reimbursed SGD 5.00 (approximately USD 3.75) for each friend successfully referred and who had completed the baseline survey; a total of 171 (30.0%) of participants were recruited through referrals. The response rate of the survey could not be established as it was not possible to ascertain the total number of eligible participants reached through the team's marketing efforts. However, we were able to calculate the completion rate of the survey among those who were eligible and enrolled in the study.

### Variable measures

The survey collected sociodemographic information from respondents. We collected age in years from participants, which was analyzed as a continuous variable. Ethnicity was collected through a categorical variable that aligned with Singapore's official racial categories of “Chinese” (*n* = 478; 83.9%), “Malay” (*n* = 45; 7.9%), “Indian” (*n* = 24; 4.2%), and “Others” (*n* = 23; 4.0%), and was then recoded into “Chinese” and “Non-Chinese.” Gender was collected through a categorical variable where participants could indicate if they were “cisgender male (assigned gender at birth is male, gender identity is male)” (*n* = 525; 92.1%), “transgender male (assigned gender at birth is not male, gender identity is male)” (*n* = 11; 1.9%), or “queer (assigned gender at birth is male, and you do not identify with any particular gender now)” (*n* = 34; 6.0%). Sexual orientation was represented as a categorical variable wherein participants indicated if they identified as “gay” (*n* = 408; 71.6%), “bisexual” (*n* = 148; 26.0%), “queer” (*n* = 12; 2.1%), or “others” (*n* = 2; 0.4%); this was then recoded into “gay” and “bisexual, queer, or others.”

Housing type was collected as a categorical variable that aligned with Singapore's public housing size categories, including “one-room public housing flats” (*n* = 5; 0.9%), “two-room public housing flats” (*n* = 9; 1.6%), “three-room public housing flats” (*n* = 69; 12.1%), “four-room public housing flats” (*n* = 182; 31.9%), “five-room public housing flats” (*n* = 142; 24.9%), “executive public housing flats” (*n* = 37; 6.5%), alongside other private housing options, such as “condominiums” (*n* = 80; 14.0%), “landed houses” (*n* = 42; 7.4%), and other private housing (*n* = 4, 0.7%). These were recoded into “public housing” and “private housing.”

The outcome variables of interest—past suicidal ideation and past suicide attempts—were collected as categorical responses where participants were asked to select either “yes,” “no,” or “prefer not to say” to the following questions: “Have you ever had thoughts of attempting suicide (i.e., suicide ideation)?” and “Have you ever attempted suicide?” respectively. This was then recoded as “yes” and “no”; participants who chose “prefer not to say” for past suicidal ideation (*n* = 47; 8.2%) or attempts (*n* = 36; 6.3%) were excluded from our analyses. Our diagnostic statistical tests indicated that participants who reported “prefer not to say” were likely reporting as such so as to not recall past traumatic experiences of suicide, as they were more similar to those who responded “yes” than those who responded “no” when comparing scores for factors that were associated with suicide-related behaviors (e.g., depression severity). However, we opted to exclude them instead of recoding them as “yes” to ensure that we did not overstate the relationship between experienced homophobia and suicide-related behaviors in our study. Supplementary analyses on varying treatments of the “prefer not to say” group in our final analyses also support the assumption above (Supplementary Tables S2 and S3).

The main covariate of interest, experienced homophobia, was a 14-item scale developed by Ramirez-Valles, et al.^28^ It assesses the degree to which individuals experienced stigma and discrimination based on their sexual orientation growing up and in adulthood. Each item was measured on a four-point Likert scale ranging from 1 to 4, with 1 being never and 4 being many times; Cronbach's alpha was 0.90.

Depression severity was measured by using the nine-item Patient Health Questionnaire-9 (PHQ-9).^29,30^ Participants were asked “over the last 2 weeks, how often have you been bothered by any of the following problems?” for a total of nine statements, to which they could respond to four possible answers on a Likert scale with 0 being not at all and 3 being nearly every day. Depression severity was measured as an index that was the sum score of all nine items, with a minimum score of 0 and a maximum score of 27. Cronbach's alpha of the scale was reported as 0.92. Self-esteem was measured by the use of a single-item self-esteem scale validated by Robins et al.^31^ Participants were asked to respond to the statement “I have high self-esteem” on a seven-point Likert scale: 1 being very untrue of me and 7 being very true of me.

Outness was measured through the outness inventory, a 10-item scale developed by Mohr and Fassinger.^32^ This scale does not assess outness with respect to gender identity. Specifically, the outness inventory assesses the degree or magnitude to which lesbian, gay, and bisexual individuals are open or “out” about their sexual orientation to other individuals. Questions asked in this scale required participants to think about how different individuals in their own social networks (e.g., mother, siblings, religious leaders) may know about, or openly talk about the participant's own sexual orientation. Participants could select responses from 1 to 7, with 1 being that the person definitely does not know about your sexual orientation and 7 being that the person definitely knows about your sexual orientation, and it is openly talked about. The overall outness score was calculated as an average of three subscales, including outness to family, outness to religion, and outness to the world; Cronbach's alpha of the scale was reported as 0.82.

### Statistical analysis

Statistical analysis was conducted using the statistical software STATA version 15 (StataCorp LLC, College Station, TX). We employed descriptive statistics to identify trends in sample characteristics, while bivariate and multivariable logistic regression models were used to compute the crude odds ratio (OR) and adjusted odds ratio (aOR) for past suicidal ideation and suicide attempts among participants. Key demographic variables, including age, ethnicity, gender, sexual orientation, and housing type were input into the multivariable analyses, alongside other variables that were statistically significant at the bivariable level. We employed STATA's sem function to generate total, direct, and indirect effects and determine the mediating effects of outness, depression severity, and self-esteem on the relationship between experienced homophobia and suicidal ideation and suicide attempts. Statistical significance was set at *p* < 0.05.

## Results

### Sociodemographic attributes and description of the analytic sample

A total of 893 participants were initially enrolled at the study baseline, and 570 participants completed the baseline survey, thus providing a completion rate of 63.8%. Table 1 summarizes the participants' characteristics in the analytic sample. The mean age was 21.9 (standard deviation [SD] = 2.17) among participants, and most identified as ethnic Chinese (*n* = 478; 83.9%), cisgender male (*n* = 525; 92.1%), gay (*n* = 408; 71.6%), and stayed in public housing (*n* = 444; 77.9%). A total of 58.9% (*n* = 308) of participants reported ever contemplating suicide, whereas 14.2% (*n* = 76) reported ever attempting suicide. Participants reported mean or median scores of 25.0 (interquartile range [IQR] = 12.0), 7.0 (IQR = 10.0), 4.1 (SD = 1.66), and 2.3 (IQR = 2.0) for experienced homophobia, depression severity, self-esteem, and outness inventory scores, respectively.

**Table 1.** Sociodemographic Attributes and Description of the Analytic Sample (*n* = 570)

Demographic variables	n	%	Mean	SD
Age			21.9	2.17
Ethnicity				
Chinese	478	83.9		
Non-Chinese	92	16.1		
Gender identity				
Cisgender male	525	92.1		
Transgender male	11	1.9		
Queer male	34	6.0		
Sexual orientation				
Gay	408	71.6		
Bisexual, queer, or others	162	28.4		
Housing type				
Private housing	126	22.1		
Public housing	444	77.9		
Ever contemplated suicide (*n* = 523)				
Yes	308	58.9		
No	215	41.1		
Ever attempted suicide (*n* = 534)				
Yes	76	14.2		
No	458	85.8		
Experienced homophobia^a^ (range: 14 to 56)			25.0	12.00
Depression severity^a^ (range: 0 to 27)			7.0	10.00
Self-esteem (range: 1 to 7)			4.1	1.66
Outness inventory^a^ (range: 1 to 7)			2.3	2.00

^a^ Median and interquartile range are reported; otherwise, mean and standard deviation are reported.

SD, standard deviation.

### Factors associated with a history of suicidal ideation

Table 2 summarizes the results of the multivariable logistic regression models for ever contemplating or attempting suicide. At the bivariable level, experienced homophobia (OR = 1.08, 95% confidence interval [CI]: 1.05–1.10), depression severity (OR = 1.16, 95% CI: 1.12–1.20), and outness (OR = 1.20, 95% CI: 1.06–1.37) were positively associated, whereas age (OR = 0.92, 95% CI: 0.85–1.00) and self-esteem (OR = 0.77, 95% CI: 0.69–0.86) were negatively associated with a history of suicidal ideation. At the multivariable level, analyses revealed that after controlling for all covariates in the model, experienced homophobia (aOR = 1.05, 95% CI: 1.02–1.07) and depression severity (aOR = 1.13, 95% CI: 1.09–1.17) were positively associated with a history of suicidal ideation.

**Table 2.** Multivariable Logistic Regression for Ever Contemplating Suicide and Attempting Suicide

	Ever contemplated suicide (*n* = 520)				Ever attempted suicide (*n* = 530)			
	OR	95% CI	aOR	95% CI	OR	95% CI	aOR	95% CI
Age	0.92^*^	**0.85–1.00**	0.96	0.87–1.05	0.93	0.83–1.03	0.96	0.85–1.08
Non-Chinese (ref. = Chinese)	1.43	0.88–2.32	1.16	0.65–2.04	1.82^*^	**1.02–3.24**	1.47	0.75–2.88
Gender identity								
Cisgender male	Ref.		Ref.		Ref.		Ref.	
Transgender male	7.30	0.93–57.44	4.54	0.54–38.46	1.58	0.33–7.58	0.98	0.18–5.24
Queer male	1.38	0.60–3.15	0.88	0.34–2.27	1.80	0.70–4.63	1.24	0.45–3.41
Gay (ref. = bisexual, queer, or others)	1.04	0.71–1.53	1.15	0.72–1.83	0.84	0.50–1.42	0.72	0.40–1.29
Private housing (ref. = public housing)	0.87	0.57–1.31	0.96	0.60–1.53	0.99	0.55–1.78	1.00	0.52–1.90
Experienced homophobia	1.08^***^	**1.05–1.10**	1.05^**^	**1.02–1.07**	1.06^***^	**1.03–1.10**	1.03	1.00–1.07
Depression severity	1.16^***^	**1.12–1.20**	1.13^***^	**1.09–1.17**	1.11^***^	**1.07–1.15**	1.08^***^	**1.04–1.12**
Self-esteem	0.77^***^	**0.69–0.86**	0.92	0.80–1.05	0.84^*^	**0.73–0.97**	0.91	0.76–1.09
Outness	1.20^**^	**1.06–1.37**	1.15	0.99–1.34	1.38^***^	**1.17–1.61**	1.40^**^	**1.15–1.69**

Statistically significant results (*p* < 0.05) are in bold font; ^*^p < 0.05, ^**^p < 0.01, ^***^p < 0.001.

aOR, adjusted odds ratio; CI, confidence interval; OR, odds ratio; Ref., reference category.

### Factors associated with a history of attempting suicide

At the bivariable level, being non-Chinese (OR = 1.82, 95% CI: 1.02–3.24), having experienced homophobia (OR = 1.06, 95% CI: 1.03–1.10), depression severity (OR = 1.11, 95% CI: 1.07–1.15), and outness (OR = 1.38, 95% CI: 1.17–1.61) were positively associated with a history of attempting suicide. Conversely, self-esteem (OR = 0.84, 95% CI: 0.73–0.97) was negatively associated with a history of attempting suicide. At the multivariable level, analyses revealed that after controlling for all covariates in the model, depression severity (aOR = 1.08, 95% CI: 1.04–1.12), and outness (aOR = 1.40, 95% CI: 1.15–1.69) were positively associated with a history of attempting suicide.

### Mediation analyses

**FIG. 1.**
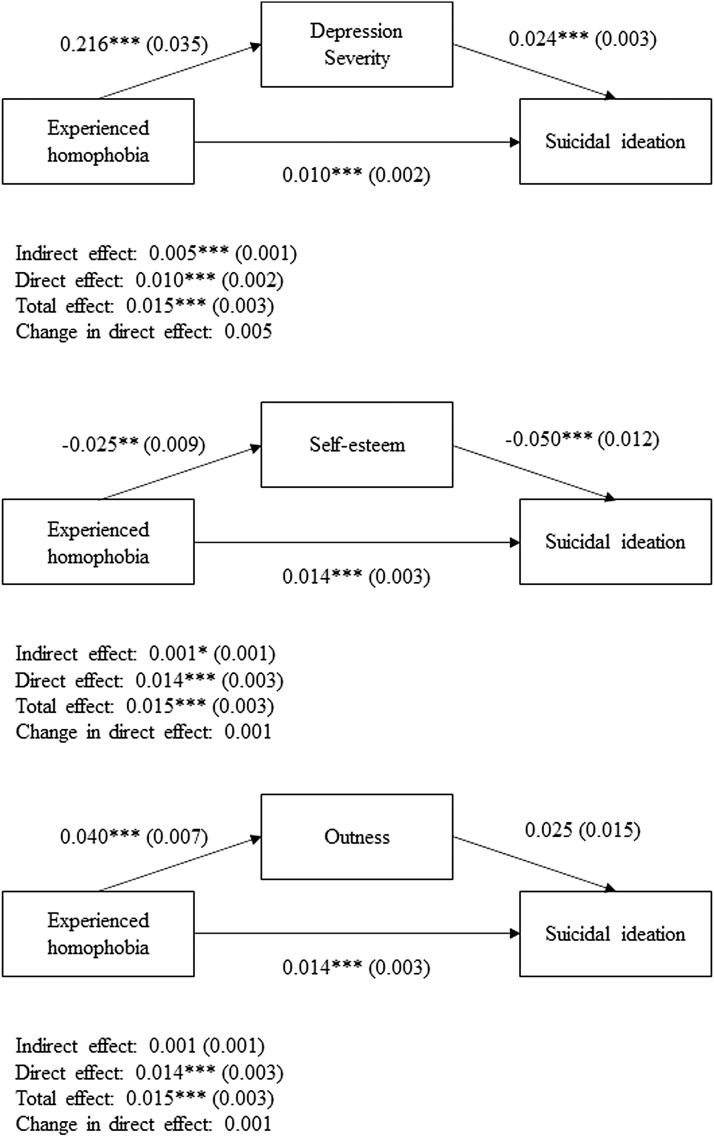
Mediation analyses for suicidal ideation. Indirect, direct, total, and changes in direct effects are reported, alongside the standard errors in parentheses. *p < 0.05, **p < 0.01, ***p < 0.001. Key demographic variables, including age, ethnicity, gender identity, sexual orientation, and housing type were added as covariates to all mediation models.

**FIG. 2.**
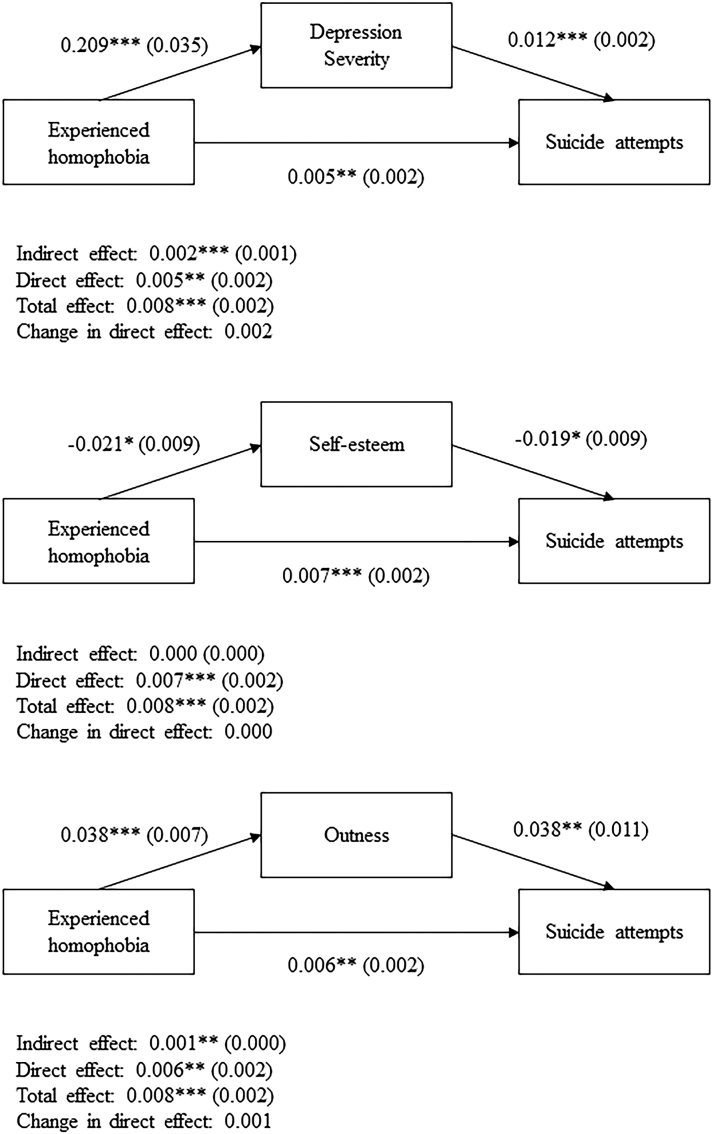
Mediation analyses for suicide attempts. Indirect, direct, total, and changes in direct effects are reported, alongside the standard errors in parentheses. *p < 0.05, **p < 0.01, ***p < 0.001. Key demographic variables, including age, ethnicity, gender identity, sexual orientation, and housing type were added as covariates to all mediation models.

Figures 1 and 2 illustrate the mediation analyses for ever reporting suicidal ideation or suicide attempts, respectively. The total, direct, and indirect effects alongside the change in direct effects are reported for each mediation model. Age, ethnicity, gender identity, sexual orientation, and housing type were included as covariates for each model. Mediation analysis revealed that depression severity (indirect effect: Coeff = 0.005, *p* < 0.001) and self-esteem (indirect effect: Coeff = 0.001, *p* < 0.05) partially mediated the effects of experienced homophobia on a history of suicidal ideation, whereas depression severity (indirect effect: Coeff = 0.002, *p* < 0.001) and outness (indirect effect: Coeff = 0.001, *p* < 0.01) partially mediated the effects of experienced homophobia on a history of attempting suicide.

## Discussion

This study is the first published study on suicide-related behaviors among young GBTQ men in Singapore. This study also sought to delineate the associations between experienced homophobia and a history of suicidal ideation and suicide attempts, as well as investigate the mediating roles of depression severity, self-esteem, and outness that account for this relationship, as part of the minority stress pathway. Our sample of young GBTQ men reported a 58.9% (*n* = 308) and 14.2% (*n* = 76) prevalence of having histories of suicidal ideation, and attempting suicide, respectively. To our knowledge, this is the first published study to measure the prevalence of such suicide-related behavior in young GBTQ men in Singapore. A nationally representative study among Singaporeans conducted from 2009 to 2010 found that 43.6% and 12.3% of those with major depressive disorders reported a history of suicidal ideation and attempting suicide,^33^ thus potentially situating our sample of young GBTQ men at greater risk of suicidal ideation and suicide attempts. The prevalence of suicidal ideation among our sample is relatively higher than those among MSM across the world, which ranged from 13.2% to 55.8% across varying settings as reported by a meta-analysis on the prevalence of suicidal ideation among MSM.^34^

Other studies in Asia have found similar trends with regard to suicidal ideation and suicide attempts among sexual minority men. A large study among adolescents in China found that same sex-attracted (21.6%) and both sex-attracted (34.7%) males reported a higher prevalence of suicidal ideation in the past year compared with their heterosexual counterparts (14.5%).^7^ A similar trend was found with regard to suicide attempts by both same-sex attracted (6.9%) and both-sex attracted (12.2%) male adolescents, compared with heterosexual male adolescents (2.2%).^7^ Another recent study among gay and bisexual men in Taiwan found that 31.0% of respondents reported suicidal ideation in the preceding year.^16^ There might be reasons to believe that the less favorable sociolegal climate in Singapore, compared with other Asian counterparts such as Hong Kong or Taiwan, might give rise to greater levels of homophobia and thus suicide-related behaviors. However, given that our survey measured lifetime prevalence of suicidal ideation and suicide attempts, we do not have sufficient evidence to determine if GBTQ men in Singapore are at greater risk of suicide than their counterparts in other Asian countries, and further research is warranted.

Experienced homophobia was also found to be a factor that was positively associated with a history of suicidal ideation among our sample. Other studies have corroborated this finding by showing how a history of suicidal ideation and suicide attempts has been associated with ever being subjected to general and sexual orientation-based violence or victimization,^35,36^ which aligns with and corroborates the minority stress model.^27^ Hatzenbuehler extends this framework by reviewing the literature and subsequently proposing a psychological mediation framework to explain how these minority stressors and stigma may lead to elevations in “emotion dysregulation, social/interpersonal problems, and cognitive processes conferring risk for psychopathology” and thus serve to mediate the impact of stigma on the incidence and development of such psychopathologies.^37^ Our findings on the mediating role of depression and self-esteem in the relationship between experienced homophobia and suicidal ideation, as well as depression on past suicide attempts thus align with, and corroborate these frameworks.

A noteworthy finding was that outness, or the extent of sexual orientation disclosure, partially mediated the effect of experienced homophobia on a history of suicide attempts. Outness was also statistically significantly positively associated with suicide attempts after controlling for other potential confounders. Although the literature has generally suggested that gay men or MSM who conceal their sexual identities and who report higher levels of internalized homophobia report worse health outcomes,^38–40^ and that an inverse relationship might exist between outness and internalized homophobia,^41^ interpretations of these relationships should be further nuanced as past studies have also found that those who are more out do not necessarily report better wellbeing.^42^ Given that identifying as GBTQ may be a concealable stigmatized identity,^43^ a possible explanation would be that outness may only be beneficial in supportive environments, and conversely expose an individual to further stigma and discrimination, which may be higher in Singapore where considerable stigma and the criminalization of sexual relations between men prevail.

### Limitations

We are mindful of several limitations of our study. The findings of this study suggest pathways between experienced homophobia and suicide-related behaviors, but are not conclusive. This is because present measures of depression severity, self-esteem, and outness were employed in analyses in contrast to past suicide-related behaviors, and thus temporality where experienced homophobia precedes both mediating factors and the suicide-related outcomes cannot be established. Prospective cohort studies among younger, adolescent GBTQ men are required instead. Furthermore, there may be selection bias in the sample, as those who are more “out” or comfortable with their sexual orientation are more likely to participate in the study, biasing our results toward the null. Information on the venues through which participants were recruited would have allowed us to hypothesize the direction of such bias, as those who are less “out” might have been less likely to have been recruited through physical venues but instead, through online links. However, such information was not collected in this study. We also acknowledge that due to the small group size of several demographic groups, such as participants who identified as transgender male (*n* = 11), the findings involving such categories should be interpreted with caution. Nevertheless, we opted to include these participants in our analyses to ensure that their responses are reflected in our study as well. Lastly, there may be cultural or societal variables, such as perceptions of culture, value frameworks, or religiosity, which were not adjusted for in our analysis that may confound the relationship between experienced homophobia and suicidal ideation.

## Conclusion

This study explored associations between experienced homophobia and suicide-related behaviors, and sought to identify the psychological factors that account for such a relationship. Our findings have clear implications for policymakers at multiple levels, which we summarize in Table 3. First, at the individual level, psychological interventions addressing experienced homophobia, minority stress, sexual identity issues, and other underlying psychological factors should be rolled out in schools and in communities as such issues and experiences of stigma may emerge early in life. At the interpersonal level, interventions should generate awareness of signs associated with or preceding suicide-related behaviors and equip individuals with skills to link at-risk individuals to the relevant support structures. At the community level, campaigns may aim to reduce sexual orientation-based stigma in the general public, and endeavor to further develop more community-based resources to tackle homophobia and other forms of sexual orientation-based stigma and violence. At the organizational and institutional level, antibullying and antidiscrimination legislation and policies based on sexual orientation may be implemented in schools and workplaces. Finally, at the public policy level, the government can enshrine some of these antidiscrimination policies into laws, and also work on the decriminalization of same-sex relations between men to reduce stigma toward sexual minority men.

**Table 3.** Recommendations to Address Experienced Homophobia

Level of influence	Recommendations for proposed and potential interventions
Individual	Psychological interventions addressing experienced homophobia, minority stress, sexual identity issues, and other underlying psychological factors.
Interpersonal	Interventions that generate awareness of signs associated with or preceding suicide-related behaviors
	Equipping individuals with skills to link at-risk individuals to the relevant support structures.
Community	Campaigns to reduce sexual orientation-based stigma in the general public.
	Developing more community-based resources to tackle homophobia and other forms of sexual orientation-based stigma and violence.
Organizational and institutional	Antibullying policies based on sexual orientation in schools.
	Antidiscrimination policies based on sexual orientation at places of work.
Public policy	Enshrining antidiscrimination policies into law.
	Decriminalization of same-sex relations between men to reduce stigma toward sexual minority men.

## Supplementary Material

Supplemental data

Supplemental data

Supplemental data

Supplemental data
