# Supplementary material for: Experienced Homophobia and Suicide Among Young Gay, Bisexual, Transgender, and Queer Men in Singapore: Exploring the Mediating Role of Depression Severity, Self-Esteem, and Outness in the Pink Carpet Y Cohort Study
Source: LGBT Health. 2021 Jun 30;8(5):349–58. doi: 10.1089/lgbt.2020.0323 (PMC8252908; doi:10.1089/lgbt.2020.0323)
Supplement: Supplemental data [file Supp_TableS1.docx]

**Supplementary Table S1. Enrolment Survey for the Study**

| **#** | **Question** | **Response categories** |
| --- | --- | --- |
| **Thank you for your interest in participating in the Pink Carpet Y Cohort Study.**  You will now be asked a total of 5 screening questions. This enrollment survey will take less than 1 minute to complete.  The results of this enrollment questionnaire will only be accessible by a staff member at Action for AIDS Singapore. This is done to safeguard your identity while you participate in this study. | | |
|  | What is your date of birth? | DD / MM / YYYY |
|  | What is your gender? | Cisgender Male (assigned gender at birth is male, gender identity is male)  Transgender Male (assigned gender at birth is not male, gender identity is male)  Queer (assigned gender at birth is male, and you do not identify with any particular gender now)  Others (please specify): ___________________ |
|  | What sexual orientation do you identify yourself with the most? | Gay  Bisexual  Queer  Straight / Heterosexual  Others (please specify): ___________________ |
|  | What is your HIV status? | HIV-negative  HIV-positive  I do not know my HIV status |
|  | What is your residence status? | Singaporean citizen  Singapore permanent resident  Others (please specify): ________________________ |

We opted to recruit participants who identified, in terms of gender identity, as cisgender male (assigned gender at birth is male, gender identity is male), transgender male (assigned female at birth, gender identity is male), or genderqueer men (assigned male at birth, do not identify with any particular gender now). This was done following consultation with the partner community group, Action for AIDS Singapore, to capture a sample that was likely to be most at risk for HIV and other sexually transmitted infections that epidemiologically was more prevalent within the Singapore gay, bisexual, and queer male community.

Supplementary Tables S2 and S3 detail how recoding “prefer not to say” as “no”, “yes”, and removing it have an impact on the final results for the present study. Our diagnostic statistical tests indicated that participants who reported “prefer not to say” were likely reporting as such so as to not recall past traumatic experiences of suicide, as they were more similar to those who responded “yes” than those who responded “no” when comparing scores for factors that were associated with suicide-related behaviors (e.g., depression severity). However, we opted to exclude them instead of recoding them as “yes” to ensure that we did not overstate the relationship between experienced homophobia and suicide-related behaviors in our study.
