# Supplementary figures and images for: Experienced Homophobia and Suicide Among Young Gay, Bisexual, Transgender, and Queer Men in Singapore: Exploring the Mediating Role of Depression Severity, Self-Esteem, and Outness in the Pink Carpet Y Cohort Study
Source: LGBT Health. 2021 Jun 30;8(5):349–58. doi: 10.1089/lgbt.2020.0323 (PMC8252908; doi:10.1089/lgbt.2020.0323)

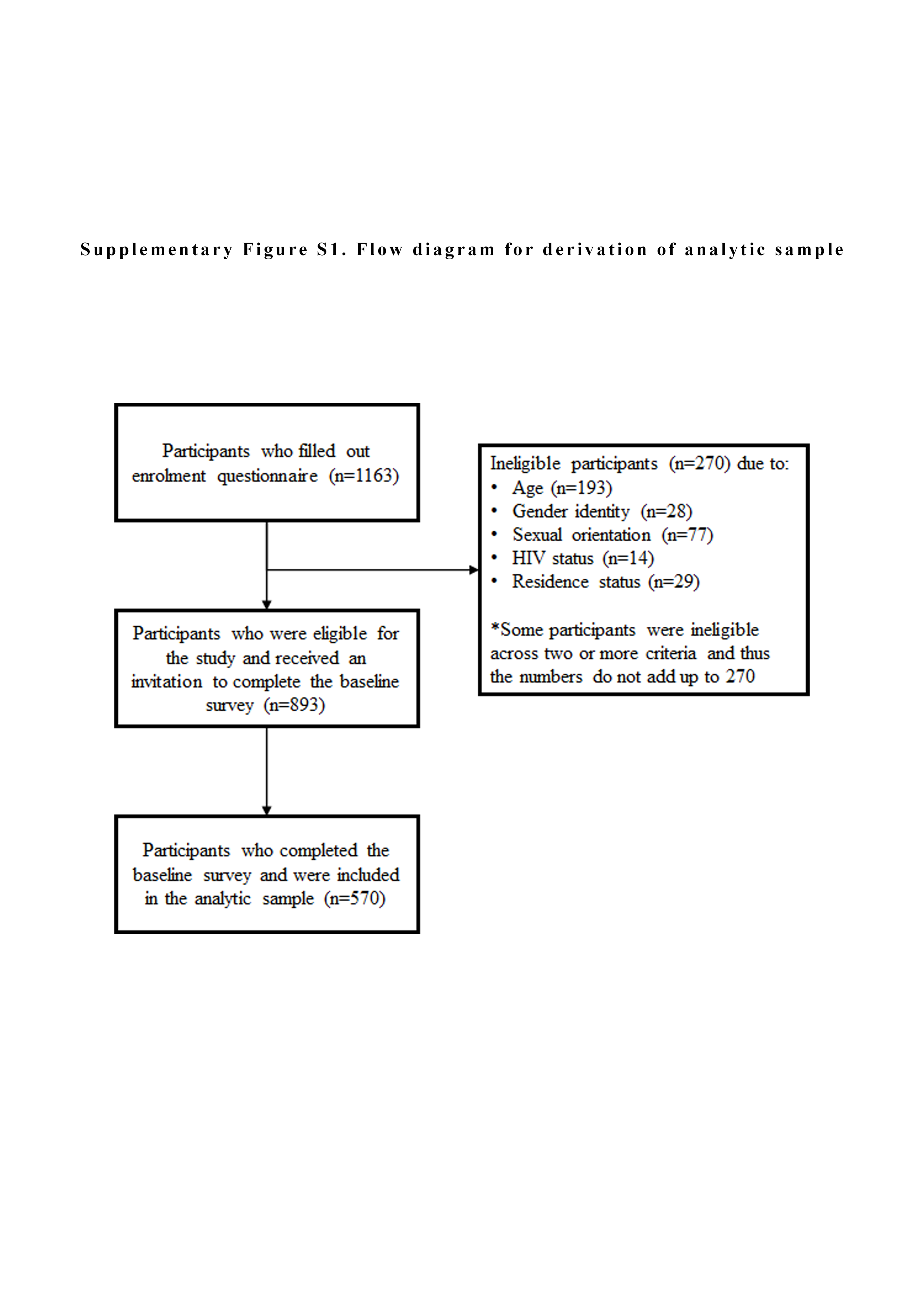

Supplement: Supplemental data [file Supp_FigS1.tif]
