# Supplementary material for: Experienced Homophobia and Suicide Among Young Gay, Bisexual, Transgender, and Queer Men in Singapore: Exploring the Mediating Role of Depression Severity, Self-Esteem, and Outness in the Pink Carpet Y Cohort Study
Source: LGBT Health. 2021 Jun 30;8(5):349–58. doi: 10.1089/lgbt.2020.0323 (PMC8252908; doi:10.1089/lgbt.2020.0323)
Supplement: Supplemental data [file Supp_TableS2.docx]

| **Supplementary Table S2. Multivariable logistic regression for ever contemplating suicide for various recodes of “prefer not to say” response** | | | | | | | | | | | |  | |
| --- | --- | --- | --- | --- | --- | --- | --- | --- | --- | --- | --- | --- | --- |
|  |  | **Ever contemplated suicide** | | | | | | | | | | |  |
|  |  | **Model 1 (n=566)** | | |  | **Model 2 (n=520)** | | |  | **Model 3 (n=566)** | | |  |
|  |  | **aOR** | **95% CI** | **p** |  | **aOR** | **95% CI** | **p** |  | **aOR** | **95% CI** | **p** |  |
| Age |  | 0.94 | (0.87-1.03) | 0.175 |  | 0.96 | (0.87-1.05) | 0.366 |  | 0.97 | (0.88-1.06) | 0.455 |  |
| Non-Chinese (ref.=Chinese) | | 1.28 | (0.76-2.16) | 0.345 |  | 1.16 | (0.65-2.04) | 0.615 |  | 1.09 | (0.62-1.91) | 0.762 |  |
| Gender Identity | |  |  |  |  |  |  |  |  |  |  |  |  |
|  | Cisgender male | Ref. |  |  |  | Ref. |  |  |  | Ref. |  |  |  |
|  | Transgender male | 6.17 | (0.74-51.63) | 0.093 |  | 4.54 | (0.54-38.46) | 0.165 |  | 3.66 | (0.43-31.08) | 0.234 |  |
|  | Queer male | 0.54 | (0.25-1.16) | 0.112 |  | 0.88 | (0.34-2.27) | 0.787 |  | 1.10 | (0.45-2.68) | 0.833 |  |
| Gay (ref.=Bisexual, queer, or others) | | 1.14 | (0.75-1.74) | 0.541 |  | 1.15 | (0.72-1.83) | 0.561 |  | 1.08 | (0.69-1.69) | 0.747 |  |
| Private housing (ref.=Public housing) | | 1.07 | (0.69-1.67) | 0.756 |  | 0.96 | (0.60-1.53) | 0.861 |  | 0.91 | (0.58-1.45) | 0.695 |  |
| Experienced homophobia | | **1.02** | **(1.00-1.05)** | **0.049** |  | **1.05** | **(1.02-1.07)** | **0.001** |  | **1.05** | **(1.02-1.08)** | **<0.001** |  |
| Depression severity | | **1.10** | **(1.07-1.14)** | **<0.001** |  | **1.13** | **(1.09-1.17)** | **<0.001** |  | **1.13** | **(1.09-1.17)** | **<0.001** |  |
| Self-esteem | | 0.94 | (0.83-1.06) | 0.317 |  | 0.92 | (0.80-1.05) | 0.223 |  | 0.88 | (0.77-1.01) | 0.070 |  |
| Outness | | **1.21** | **(1.05-1.40)** | **0.007** |  | 1.15 | (0.99-1.34) | 0.061 |  | 1.14 | (0.98-1.32) | 0.082 |  |
|  |  |  |  |  |  |  |  |  |  |  |  |  |  |
|  | |  |  |  |  |  |  |  |  |  |  |  |  |
| CI, confidence interval; Ref., reference category; OR, odds ratio; aOR, adjusted odds ratio. | | | | | | | | |  |  |  |  | |
| Statistically significant results (p<0.05) are highlighted in bold font. | | | | | | | | | | | | |  |
| Model 1 recodes all participants who indicated “prefer not to say” to the question on ever contemplating suicide as “no”. | | | | | | | | | |  |  |  | |
| Model 2 excludes all participants who indicated “prefer not to say” to the question on ever contemplating suicide. | | | | | | | | |  |  |  |  | |
| Model 3 recodes all participants who indicated “prefer not to say” to the question on ever contemplating suicide as “yes”. | | | | | | | | | |  |  |  | |
