# Supplementary material for: Experienced Homophobia and Suicide Among Young Gay, Bisexual, Transgender, and Queer Men in Singapore: Exploring the Mediating Role of Depression Severity, Self-Esteem, and Outness in the Pink Carpet Y Cohort Study
Source: LGBT Health. 2021 Jun 30;8(5):349–58. doi: 10.1089/lgbt.2020.0323 (PMC8252908; doi:10.1089/lgbt.2020.0323)
Supplement: Supplemental data [file Supp_TableS3.docx]

| **Supplementary Table S3. Multivariable logistic regression for ever attempting suicide for various recodes of “prefer not to say” response** | | | | | | | | | | | |  |
| --- | --- | --- | --- | --- | --- | --- | --- | --- | --- | --- | --- | --- |
|  |  | **Ever attempted suicide** | | | | | | | | | | |
|  |  | **Model 1 (n=566)** | | |  | **Model 2 (n=530)** | | |  | **Model 3 (n=566)** | | |
|  |  | **aOR** | **95% CI** | **p** |  | **aOR** | **95% CI** | **p** |  | **aOR** | **95% CI** | **p** |
| Age | | 0.95 | (0.84-1.07) | 0.380 |  | 0.96 | (0.85-1.08) | 0.495 |  | 1.01 | (0.91-1.12) | 0.832 |
| Non-Chinese (ref.=Chinese) | | 1.74 | (0.91-3.35) | 0.094 |  | 1.47 | (0.75-2.88) | 0.256 |  | 0.97 | (0.52-1.80) | 0.915 |
| Gender identity | |  |  |  |  |  |  |  |  |  |  |  |
|  | Cisgender male | Ref. |  |  |  | Ref. |  |  |  | Ref. |  |  |
|  | Transgender male | 0.75 | (0.14-4.09) | 0.740 |  | 0.98 | (0.18-5.24) | 0.977 |  | 0.89 | (0.21-3.84) | 0.877 |
|  | Queer male | 0.94 | (0.35-2.50) | 0.899 |  | 1.24 | (0.45-3.41) | 0.678 |  | 1.84 | (0.83-4.07) | 0.134 |
| Gay (ref.=Bisexual, queer, or others) | | 0.72 | (0.40-1.30) | 0.277 |  | 0.72 | (0.40-1.29) | 0.271 |  | 0.73 | (0.43-1.22) | 0.230 |
| Private housing (ref.=Public housing) | | 1.05 | (0.56-1.98) | 0.878 |  | 1.00 | (0.52-1.90) | 0.991 |  | 0.95 | (0.54-1.68) | 0.873 |
| Experience homophobia | | 1.02 | (0.99-1.05) | 0.290 |  | 1.03 | (1.00-1.07) | 0.055 |  | **1.05** | **(1.03-1.08)** | **<0.001** |
| Depression severity | | **1.08** | **(1.03-1.12)** | **<0.001** |  | **1.08** | **(1.04-1.12)** | **<0.001** |  | **1.07** | **(1.03-1.11)** | **<0.001** |
| Self-esteem | | 0.91 | (0.77-1.09) | 0.309 |  | 0.91 | (0.76-1.09) | 0.326 |  | **0.86** | **(0.74-0.99)** | **0.042** |
| Outness | | **1.42** | **(1.17-1.71)** | **<0.001** |  | **1.40** | **(1.15-1.69)** | **0.001** |  | **1.32** | **(1.11-1.56)** | **0.001** |
|  |  |  |  |  |  |  |  |  |  |  |  |  |
|  | | | | | | | | | | |  |  |
| Statistically significant results (p<0.05) are highlighted in bold font. | | | | |  |  |  |  |  |  |  |  |
| Model 1 recodes all participants who indicated “prefer not to say” to the question on ever attempting suicide as “no”. | | | | | | | | | | |  |  |
| Model 2 excludes all participants who indicated “prefer not to say” to the question on ever attempting suicide. | | | | | | | | | |  |  |  |
| Model 3 recodes all participants who indicated “prefer not to say” to the question on ever attempting suicide as “yes”. | | | | | | | | | | |  |  |
